# Evaluation of the Effect of Granite Waste Powder by Varying the Molarity of Activator on the Mechanical Properties of Ground Granulated Blast-Furnace Slag-Based Geopolymer Concrete

**DOI:** 10.3390/polym14020306

**Published:** 2022-01-13

**Authors:** Fatheali A. Shilar, Sharanabasava V. Ganachari, Veerabhadragouda B. Patil, Kottakkaran Sooppy Nisar, Abdel-Haleem Abdel-Aty, I. S. Yahia

**Affiliations:** 1 Department of Civil Engineering, Jain College of Engineering, Belagavi 590014, India; shilarone@gmail.com; 2 Department of Chemistry, School of Advanced Sciences, KLE Technological University, Hubballi 580031, India; 3 Institute of Energetic Materials, Faculty of Chemical Technology, University of Pardubice, 53210 Pardubice, Czech Republic; IamVeerabhadraa@gmail.com; 4 Department of Mathematics, College of Arts and Sciences, Prince Sattam Bin Abdulaziz University, Wadi Aldawaser 11991, Saudi Arabia; n.sooppy@psau.edu.sa; 5 Department of Physics, College of Sciences, University of Bisha, P.O. Box 344, Bisha 61922, Saudi Arabia; amabdelaty@ub.edu.sa; 6 Physics Department, Faculty of Science, Al-Azhar University, Assiut 71524, Egypt; 7 Research Center for Advanced Materials Science (RCAMS), King Khalid University, P.O. Box 9004, Abha 61413, Saudi Arabia; isyahia@gmail.com; 8 Laboratory of Nano-Smart Materials for Science and Technology (LNSMST), Department of Physics, Faculty of Science, King Khalid University, P.O. Box 9004, Abha 61413, Saudi Arabia; 9 Nanoscience Laboratory for Environmental and Biomedical Applications (NLEBA), Semiconductor Lab, Department of Physics, Faculty of Education, Ain Shams University, Roxy, Cairo 11757, Egypt

**Keywords:** granite waste powder, GGBS, alkaline to binder ratio, molarity, geopolymer concrete

## Abstract

Industrial waste such as Ground Granulated Blast-Furnace Slag (GGBS) and Granite Waste Powder (GWP) is available in huge quantities in several states of India. These ingredients have no recognized application and are usually shed in landfills. This process and these materials are sources of severe environmental pollution. This industrial waste has been utilized as a binder for geopolymers, which is our primary focus. This paper presents the investigation of the optimum percentage of granite waste powder as a binder, specifically, the effect of molar and alkaline to binder (A/B) ratio on the mechanical properties of geopolymer concrete (GPC). Additionally, this study involves the use of admixture SP-340 for better performance of workability. Current work focuses on investigating the effect of a change in molarity that results in strength development in geopolymer concrete. The limits for the present work were: GGBS partially replaced by GWP up to 30%; molar ranging from 12 to 18 with the interval of 2 M; and A/B ratio of 0.30. For 16 M of GPC, a maximum slump was observed for GWP with 60 mm compared to other molar concentration. For 16 M of GPC, a maximum compressive strength (CS) was observed for GWP with 20%, of 33.95 MPa. For 16 M of GPC, a maximum STS was observed for GWP, with 20%, of 3.15 MPa. For 16 M of GPC, a maximum FS was observed for GWP, with 20%, of 4.79 MPa. Geopolymer concrete has better strength properties than conventional concrete. GPC is $13.70 costlier than conventional concrete per cubic meter.

## 1. Introduction

An interesting picture in the March 2020 issue of National Geographic magazine showed that, annually, the world economy uses more than 100,000 million tons of natural resources (an average of 13 tons per person). Of that consumption, 67,000 million tons are transformed into atmospheric pollution, such as carbon dioxide, or become solid waste, such as the plastics that enter the oceans each year. Of the remaining 35,000 million tons, only 8400 million tons are reused [1,2]. The construction materials manufacturing industry, notably the concrete sector, is having difficulty meeting market demand due to the scarcity of limestone of higher quality [3]. Concrete is responsible for 5% to 6% of global carbon emissions and there is a demand for new material to meet the expectations of the industry and customers [4]. The polymerization process involves a rapid chemical reaction on Si-Al minerals under alkaline conditions, resulting in a 3D polymeric chain and ring structure composed of Si-O-Al-O bonds [5]. Geopolymer binder is recognized as a viable alternative to cementitious material because of its inherent merits of low embodied energy and lower carbon emission into the environment [6]. Geopolymer concrete (GPC) is formulated by the activation of industrial by-products containing supplementary cementitious materials with alkaline activators [2,7]. GPC applicability is mainly confined to off-site precast and modular construction because of its curing needs and safety concerns over exposure of untrained employees to alkaline activators [8,9]. A geopolymer precursor can be any source of silica and alumina dissolved in an alkaline solution termed activating solution [10]. However, the most commonly used alumina-silicate sources are fly ash (FA). FA, ground granulated blast-furnace slag (GGBS), and metakaolin (MK) have sparked much attention because of their excellent mechanical characteristics and greater purity due to the uniformity of their chemical composition [8,11].

In the production of GPC, the chemical composition of raw material has a significant impact on strength improvement. The different chemical content of industrial waste may influence the different properties and the performance of GPC [12,13]. Granite dust is a byproduct of stone cutting and grinding. The granite granules are combined with water, thus creating a colloidal waste throughout the manufacturing process [14,15]. Granite is a classic, high-quality material widely spread throughout the earth’s oceanic crust and is the most prevalent mafic igneous rock produced from magma. This material is frequently utilized in civil engineering as an aggregate in the production of concretes or asphalt for road building. According to recent reports, the quantity of waste generated during the manufacturing phases of the granite sector accounts for about 65% of total manufacturing [16]. With reductions in landfill space and the imposition of regulatory regulations, disposal costs are rising, and companies are attempting to discover ways to reuse waste [16,17]. The manufacture and widespread use of this material create a massive volume of non-biodegradable waste [17,18]. Granite industry waste materials are estimated to be 20–25% of its global output in the form of granite waste powder (GWS), resulting in millions of tons of colloidal waste every year that cause pollution and environmental harm [19,20,21]. Granite scrap is used as an aggregate in civil engineering, although no granite powder has been documented in modern buildings [22]. Granite sawdust can cause silicosis when breathed by people and, when combined with water, it produces colloidal granite waste, contaminating land and subsurface waterways. The authors investigated the life cycle assessment of GPC and compared it to regular concrete. The author concluded that the impact made by GPC on global warming is lesser than regular concrete [23]. Due to their favorable features such as early high strength and resistance to acid and sulphate, geopolymer binding materials have shown to be a very effective sustainable choice as a replacement for cement in the construction of concrete in recent years. The reduction in carbonation depth with the inclusion of granite waste was mostly due to the high densification of the pore structure of geopolymer concrete, as finer granite waste particles effectively filled the smaller spaces between the constituent elements of geopolymer concrete [24,25]. The use of up to 20% granite debris in geopolymer concrete had a significant beneficial effect on water permeability, chloride penetration depth, and carbonation depth, all of which were significantly reduced when compared to controlled geopolymer concrete in testing. Acid assault was found to severely harm geopolymer concrete [17,26]. The acid resistance of geopolymer concrete can be improved by using 20% granite debris as natural, fine aggregates. The use of granite waste in geopolymer concrete reduces its workability, which may be effectively enhanced by using a variety of super plasticizers [27,28].

The strength development of GPC varies depending on the molar concentration, NaOH/Na_2_SiO_3_ ratio, alkaline to binder ratio (A/B), type of binder, type of activator agent, and curing condition [7,25,29]. The alkali content and Na_2_O to Al_2_O_3_ ratio contribute more efficiently to the formation of the geopolymer phase. The pH increases as the molarity increases, promoting the formation of the amorphous phase [30].

In this paper, an attempt has been made to utilize the GWP as binder content in preparation of GGBS-based GPC. GWP replaced GGBS; the effect of GWP on the fresh and mechanical properties of GPC have been evaluated. For different molar concentrations, the performances of GWP in GPC was studied. Fresh properties such as slump and mechanical properties such as CS, STS and FS was analyzed. In addition, cost estimation of GPC and its comparison with conventional concrete was carried out.

## 2. Materials Characterization and Methodology

### 2.1. Materials and Chemicals

For this study, GGBS and GWP were used as the binder in the preparation of GPC. Table 1 represents the chemical composition and physical properties of GGBS and GWP.

The elemental composition of materials may be determined using XRF (X-ray fluorescence, Rigaku, Austin, TX, USA) by the manufacturer, a non-destructive analytical method. XRF analyzers assess the chemistry of a sample by measuring the fluorescence (or secondary) X-ray generated from it when stimulated by a main X-ray source. Each element present in a sample emits a distinct set of fluorescent X-rays that are unique to that element, which is why XRF spectroscopy is an effective technique for qualitative and quantitative material composition investigation. GGBS is procured from Bellary JSW, confirmed to IS: 12,089, with a specific gravity of 2.91. GWP is the waste originating from the granite sawing process. GWP is procured from Shanti Shri, Hubballi, India, with a specific gravity of 2.59. River sand has specific gravity of 2.39 and belongs to zone II. Coarse aggregate with 20 mm downsizes with specific gravity of 2.6, as fine, and coarse aggregate confirmed to IS 383. Distilled water added to preparing GPC to make a homogeneous mix. NaOH and Na_2_SiO_3_ were purchased from Ganesh chemicals, Dharwad, India. This pellet of NaOH was dissolved in distilled water to make the required molar concentration. The activator solution was prepared one day before casting of GPCs. Molar concentration ranging from 12 to 18 M, with an interval of 2 M, alkaline to binder ratio 0.30 kept constant, super-plasticizer of SP-430 with a specific gravity of 1.25 and dosage of 2.5% were considered for the present study.

### 2.2. Methodology

After referring to various earlier literature and IS code 10262, a mixed design was prepared for GPC. The physical property of ingredients of GPC is presented in Table 2. The activator solution was prepared a day before casting [29]. While casting, binder, aggregate and activator were weighed, the binder allows mix GPC matrix uniformly. SP-430, an admixture with a dosage of 2.5% used in the preparation of GPC, was added directly into the alkaline solution and mixed. The solution was poured into the dry binder [30]. Mixing of concrete was carried out in a concrete mixer, the alkaline solution was mixed with ingredients, and GPC was prepared [31]. Three samples for each mix were cast for GPC, and average values were reproduced as results for CS, STS, FS, WA, and BD investigation.

During the fresh state, the slump test was performed. The mechanical properties such as CS, STS, FS, WA, and BD were investigated. Curing of GPC was carried out, by covering the cubes/cylinder/beam with gunny bags at ambient temperature.

## 3. Testing Method

### 3.1. Workability

Slump tests were conducted for the fresh properties of GPC to measure ease of mixing, placing, and compaction before it sets. The arrangement comprises the frustum of a cone and tamping rod confirmed to IS 1199 [32]. Before the test, the internal surface of the cone was systematically cleaned, the cone infused in four layers. After each layer of concrete was laid inside the cone, concrete in the cone was tamped with a tamping rod for 25 strokes. After completing the top layer, leveling of the concrete surface was achieved using a trowel, and the height of the cone was measured. The cone was slowly lifted vertically, and concrete height was again measured. Differences between the heights give the slump height, measured (in mm).

### 3.2. Compressive Strength Test

The compressive strength test was confirmed to be IS 516 [33]. The size of the cube mold is 150 mm^3^. The GPC filled three-layers into the mold, each layer tamped with 35 strokes using a tamping rod. Concrete cubes were kept on the table vibrator for compaction, and before the final set of concrete, they were covered with gunny bags for curing; next day cube demolded, and testing of the cube was performed. Compression testing machine (CTM, Aimil, New Delhi, India) of 2000 N capacity was used, loading rate applied to 140 kg/cm^2^/min used for crushing concrete.

### 3.3. Split Tensile Test

The split tensile test confirmed IS 5816 [34]. The size of the cylinder is 150 (dia.) × 300 (ht.) mm. CTM with the load rate applied between 1.2 to 2.4 N/(mm/min) was used to test the cylinder. The formula for STS is given below.

Fc = 2P/πLD(1)

where, π = 3.142, Fc—measured split tensile strength-maximum Load (N), L—length of the specimen, D—cross-sectional diameter of the specimen.

### 3.4. Flexural Strength Test

The flexural strength test was carried as per IS:516 [33] standards. The three-point loading approach was used, which involved cleaning the bearing surfaces of the supporting and loading rollers. The specimen size used was 100 × 100 × 500 mm. The load was applied to the topmost surface of the test specimen beam along two lines, 13.3 cm apart on the topmost surface of the beam. The specimen was subjected to a progressive load of 180 kg/cm^2^/min until it failed.

FS = PL/(B D^2^) (2)

P—Failure load of the specimen, L—Length of the specimen-Width of the specimen, D—Depth of the specimen.

### 3.5. Water Absorption Test

Water absorption test was conducted as per the ASTM C1585-20 [35]. The dry weight of the GPB specimen was noted, e.g., (A), at room temperature. GPC was immersed in clean water for 24 h at room temperature. Removed the specimen and removed the traces of water present on the surface of the GPC with a damp cloth. Weight of the specimen noted (B). Equation (3) was used to calculate the percentage of water absorption.

Water Absorption = (B − A)/A ∗ 100.(3)

### 3.6. Bulk Density Test

The bulk density of GPC was determined using Equation (4).

Bulk Density = (Weight of specimen (W))/(Volume of specimen (V))(4)

## 4. Results and Discussion

### 4.1. Effect of GWP on the Fresh State of GPC

**Figure 1:**
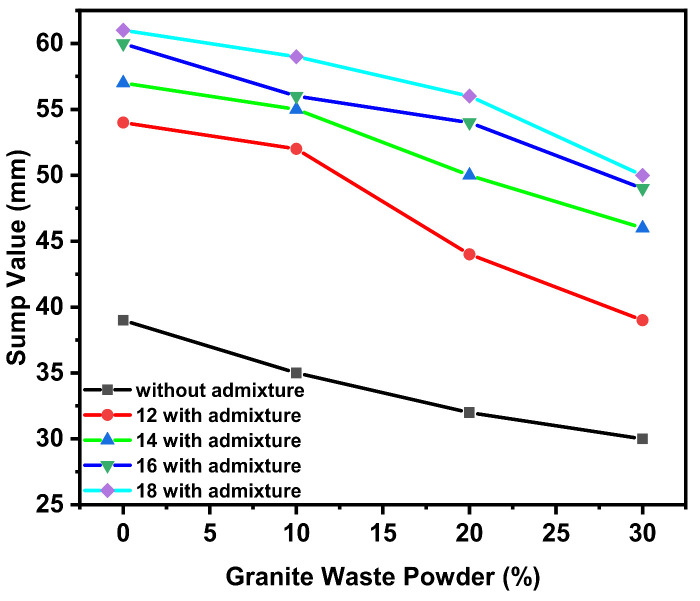
For other percent of GWP and molarity, the slump value results are shown.

The workability of GPC with river sand was measured by a workability test conducted as per IS 1199. Workability such as slump (SV) for a different proportion of GWP for various molar was carried out. The SV was observed during experimentation for various GPC-GS series, the range of slump observed was from 30 to 62 mm for various design mixes represented in Figure 1. For 12 M of GPC, maximum slump was observed for GWP with 0% (100% GGBS) with 54 mm and minimum slump observed for GWP with 30% with 41 mm. For 14 M of GPC, maximum slump observed for GWP with 0% (100% GGBS) with 57 mm and minimum slump observed for GWP with 30% with 49 mm. For 16 M of GPC, maximum slump observed for GWP with 0% (100% GGBS) with 60 mm and minimum slump observed for GWP with 30% with 53 mm. For 18 M of GPC, maximum slump observed for GWP with 0% (100% GGBS) with 62 mm and minimum slump observed for GWP with 30% with 54 mm. As the molar concentration increases, the slump value increases. It was also observed that with the increase in the GWP content in the mix, the slump value also decreased, in Figure 1.

The ratio of Na_2_SiO_3_/NaOH plays a vital role in the enhancement of slump. GWP is combined with the NaOH agent. The result is a cohesive and sticky GPC. The lower the water to binder ratio, the stronger the GPC [36]. A lower Si/Al ratio in the mix causes a slower reaction, and the slump is reduced. The slump value in GPC decreased when the ratio of Na_2_SiO_3_/NaOH increased, owing to the high viscosity of sodium silicate, which restricted the flow of mixes [37,38,39]. Because C-A-S-H was created, which decreases the slump value, higher CaO concentration in GGBS resulted in faster development of setting time. The shape of particles has more significant influences on the effect of workability of GPC [40].

In contrast, GGBS has angular particle shape; the larger surface area and high porosity of rich content of silica-based ingredients are the two factors affecting workability. The high amount of amorphous silica with porous structured particles in geopolymer paste increases the specific surface area leading to better reactivity GWP, which signifies a heterogeneous microstructure in which particles form agglomerations and become dense with a variety of particles [41,42,43,44]. GWP binders are high in Na ions, and the Na/Al molar ratio increases as fusion occurs. The reaction of GGBS with silica resulted in the formation of a new crystalline phase. GGBS alkali fusion resulted in the transformation of certain mineral phases to amorphous and new crystalline phases in the matrix. As the molar concentration of alkaline agents is increased, that increases the slump of concrete. in conventional concrete, water plays a significant role in increasing the slump and admixture, but in GPC a similar role is played by water to solids ratio and alkali/binder ratio [40,41,42,45].

### 4.2. Effect of GWP on the Hardened Properties

#### 4.2.1. Effect of GWP on the Compressive Strength (CS) of GPC

**Figure 2:**
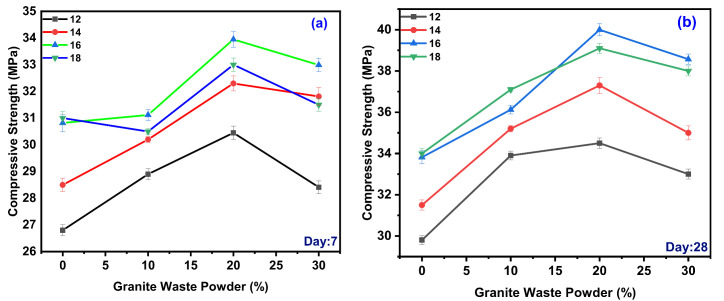
Variation of GWP on the different molarity for CS days 7 (**a**) and 28 (**b**).

Compressive strength (CS) test was conducted as per IS 516 codes in compression testing machine with 150 mm^3^ specimens. The CS values for various design mixes of geopolymer concrete for 7 and 28 days. The CS was observed during experimentation for various GPC-GS series, the range of CS observed was from 26.8 to 33.95 MPa for various design mixes for day seven represented in Figure 2a. For 12 M of GPC, maximum CS was observed for GWP with 20% with 30.45 MPa and minimum CS observed for GWP with 0% with 26.8 MPa. For 14 M of GPC, maximum CS observed for GWP with 20% with 32.3 MPa and minimum CS observed for GWP with 0% with 28.5 MPa. For 16 M of GPC, maximum CS observed for GWP with 20% with 33.95 MPa and minimum CS observed for GWP with 0% with 30.82 MPa. For 18 M of GPC, maximum slump observed for GWP with 20% with 33 MPa and minimum CS observed for GWP with 0% with 31 MPa. As the molar concentration increases, the CS increases. Concentration at 16 M shows remarkably higher CS than others. It is also observed that the increase in the GWP content in the mix increases up to 20%, beyond 20% CS declined. The CS observed during experimentation for various GPC-GS series, the range of CS observed was from 26.8 to 33.95 MPa for various design mixes for day 28 represented in Figure 2b.

For 12 M of GPC, maximum CS observed for GWP with 20% with 34.5 MPa and minimum CS observed for GWP with 0% with 29.8 MPa. For 14 M of GPC, maximum CS was observed for GWP with 20% with 37.3 MPa and minimum CS observed for GWP with 0% with 31.5 MPa. For 16 M of GPC, maximum CS was observed for GWP with 20% with 40 MPa and minimum CS observed for GWP with 0% with 33.82 MPa. For 18 M of GPC, maximum slump was observed for GWP with 20% with 39.1 MPa and minimum CS was observed for GWP with 0% with 34 MPa. As the molar concentration increases, the CS increases. However, 16 M shows remarkably higher CS than other molars. It was also observed that the increase in the GWP content in the mix increases up to 20%, beyond 20% CS decreased.

As an alkaline solution, sodium silicate solution results in more silica gel from GGBS, and GWP promotes the production of denser Si–O–Si linkages during polymerization [43,44,45,46,47,48]. The Si–O–Si link, on the other hand, is far more robust than the Si–O–Al and Al–O–Al bonds. It results in increased compressive strength. The silica and alumina in the mix binding agent are detached by the NaOH solution, strengthening the monomer bond and speeding up the polymerization process [10,33,49,50,51,52,53]. The CS value of GPC is determined by several parameters, including the molarity of the activator and the curing condition. The CS value of GPC subjected to oven-cured conditions increases with increasing molarity up to an optimal threshold beyond which CS decreases [48,49,50,51,52,53,54]. The CS value of PC subjected to oven-cured conditions increases with increasing molarity up to an optimal point beyond which the CS value decreases.

The reduction in CS, silica-rich materials adversely affects the matrix structure of geopolymer composites, which causes the formation of silica gel to be hindered; excess of silicate delays water evaporation during the polycondensation process. GGBS content is increased in GPC [46,47,48,55]. The CS also increases due to the aluminosilicate glassy nature of GGBS. When it reacts with alkaline activators and gets dissolved in it, and calcium content is increased in GPC, it increases the strength and reduces the rate of workability. GGBS substitution with GWS in minimal amounts improves cement particle dispersion in the mix, resulting in improved cement reactions and, ultimately, increases in strength and other concrete properties [20,38,43,56]. The GWS increase in reactive phases implies that the alkali fusion process resulted in physicochemical changes such as the breakage of specific crystal structures and the liberation of silica and alumina, which enhanced reactivity, leading to increase in CS [37,49,57].

#### 4.2.2. Effect of GWP on the Split Tensile Strength (STS) of GPC

**Figure 3:**
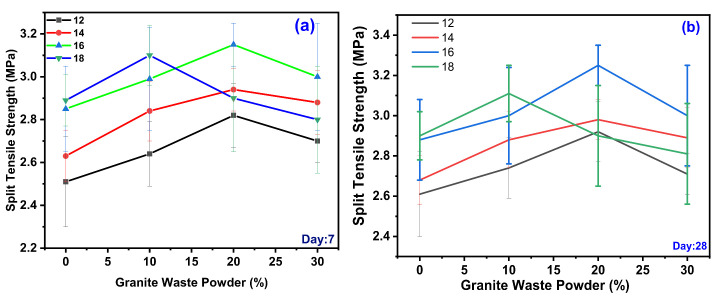
Variation of GWP on the different molarity for STS days 7 (**a**) and 28 (**b**).

The split tensile strength (STS) test was carried out as per IS 5816 and obtained results for variation design mix for day seven and day 28, shown in Figure 3. The STS was observed during experimentation for various GPC-GS series; the range of STS observed was from 2.51 to 3.15 MPa for various design mixes for day seven, represented in Figure 3a. For 12 M of GPC, maximum STS observed for GWP with 20% with 2.82 MPa and minimum STS observed for GWP with 0% with 2.51 MPa. For 14 M of GPC, maximum STS was observed for GWP with 20% with 2.94 MPa and minimum STS observed for GWP with 0% with 2.63 MPa. For 16 M of GPC, maximum STS was observed for GWP with 20% with 3.15 MPa and minimum STS observed for GWP with 0% with 2.85 MPa. For 18 M of GPC, maximum slump was observed for GWP with 10% with 3.1 MPa and minimum STS observed for GWP with 0% with 2.89 MPa. As the molar concentration increases, the STS increases; 16 M shows a remarkably higher STS than other molars. It is also observed that with an increase in the GWP content in the mix, the STS increases up to 20%, beyond 20% STS decreases.

The STS was observed during experimentation for various GPC-GS series, the range of STS observed was from 2.61 to 3.25 MPa for various design mixes for day 28 represented in Figure 3a. For 12 M of GPC, maximum STS was observed for GWP with 20% with 2.92 MPa and minimum STS observed for GWP with 0% with 2.61 MPa. For 14 M of GPC, maximum STS was observed for GWP with 20% with 2.98 MPa and minimum STS observed for GWP with 0% with 2.68 MPa. For 16 M of GPC, maximum STS was observed for GWP with 20% with 3.25 MPa and minimum STS observed for GWP with 0% with 2.88 MPa. For 18 M of GPC, maximum STS was observed for GWP with 10% with 3.11 MPa and minimum STS observed for GWP with 0% with 2.9 MPa. As the molar concentration increases, the STS increases. Similarly, 16 M shows a remarkably higher STS than other molar concentrations. It was also observed that the increase in the GWP content in the STS mix increases up to 20%, beyond 20% STS decreases.

The size, shape, and type of aggregate, the bond between binding agent and aggregate, and the bonding strength of geopolymer gel plays a significant role in developing the STS [50,51,58,59,60]. It was reported that the binding strength of geopolymer gel is interrelated to the high level of dissolution of aluminosilicates in alkaline agent presences, leading to increased geopolymerization [52,61]. The solubility rate is different for GGBS and other silica content-rich ingredients. The GWP, up to 20% in the overall mix, leads to an increase in STS, beyond 20% leads to the presence of an external impurity in the granite dust and reduces strength [53,62,63,64,65,66]. The interlocking between binding agent and aggregate is insufficient also resulting in earlier decreases in strength. It was reported that the appearance of fractures in a matrix is most likely due to the escape of free water that did not precipitate in the reaction. With increased Na content, fractures in GPC were reduced [54,55,67]

#### 4.2.3. Effect of GWP on the Flexural Strength (FS) of GPC

**Figure 4:**
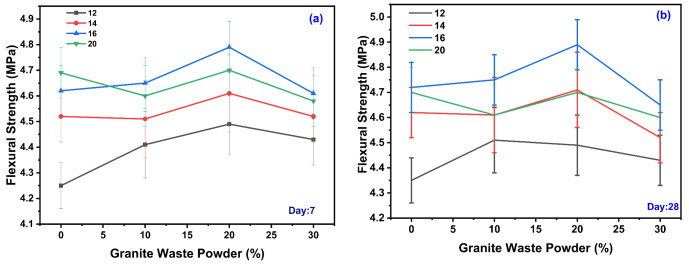
Variation of GWP on the different molarity for FS days 7 (**a**) and 28 (**b**).

Flexural strength (FS) test was carried out as per IS 516 and obtained results for variation design mix for day seven and day 28, shown in Figure 4. The FS was observed during experimentation for various GPC-GS series; the range of FS observed was from 4.25 to 4.79 MPa for various design mixes for day seven, represented in Figure 4a. For 12 M of GPC, maximum FS was observed for GWP with 20% with 4.49 MPa and minimum FS observed for GWP with 0% with 4.25 MPa. For 14 M of GPC, maximum FS was observed for GWP with 20% with 4.61 MPa and minimum FS observed for GWP with 0% with 4.52 MPa. For 16 M of GPC, maximum FS was observed for GWP with 20% with 4.79 MPa and minimum FS observed for GWP with 0% with 4.62 MPa. For 18 M of GPC, maximum FS was observed for GWP with 20% with 4.7 MPa and minimum FS was observed for GWP with 0% with 4.6 MPa. As the molar concentration increases, the FS increases. In 16 M showed a remarkably higher FS than other concentrations. It was also observed that the increase in the GWP content in the FS increased up to 20%, beyond 20% FS decreased.

The FS observed during experimentation for various GPC-GS series, the range of FS observed was from 4.35 to 4.89 MPa for various design mixes for day 28 represented in Figure 4b. For 12 M of GPC, maximum FS was observed for GWP with 20% with 4.50 MPa and minimum FS observed for GWP with 0% with 4.35 MPa. For 14 M of GPC, maximum FS was observed for GWP with 20% with 4.71 MPa and minimum FS observed for GWP with 0% with 4.62 MPa. For 16 M of GPC, maximum FS was observed for GWP with 20% with 4.89 MPa and minimum FS observed for GWP with 0% with 4.72 MPa. For 18 M of GPC, maximum FS was observed for GWP with 20% with 4.7 MPa and minimum FS observed for GWP with 0% with 4.68 MPa. As the molar concentration increases, the FS increases. However, in this case also, 16 M shows a remarkably higher FS than other molar concentrations. It was also observed that the increase in the GWP content in the mix increased up to 20%, beyond 20% FS decreases.

The FS increases as the Si/Al ratio in the mix increases to certain extent. Geopolymer concrete has superior FS to OPC concrete because of the better bonding between the geopolymer paste and aggregate [8,9,55,63,68]. The FS and STS of the geopolymer are superior to those of OPC concrete. This matrix has a greater number of unreacted GWP particles due to the significant shrinkage in the matrix, causing the decline in FS in GPC. The formation of microcracks in ITZ is another essential aspect of lower strength development [56,57,58,69]. FS specimens that cured at room temperature are unable to support their weight. A brittle mechanism of failure was discovered in beam specimens [60,64,67,70].

In comparison to ambient-cured specimens, oven-cured specimens had the highest FS value. As the molar concentration increases, the FS also increases in ambient curing conditions. Increase in the viscous activator agent results in a decrease in the unreacted particle of GGBS in the matrix. Due to this, there is strong bond development between silica and alumina ions [59,60]. The Na_2_SiO_3_/NaOH ratio is decreased, making sodium silicate less viscous than sodium hydroxide, resulting in the decrement of FS [61,62].

#### 4.2.4. Effect of GWP on the Water Absorption (WB) and Bulk Density (BD) of GPC

**Figure 5:**
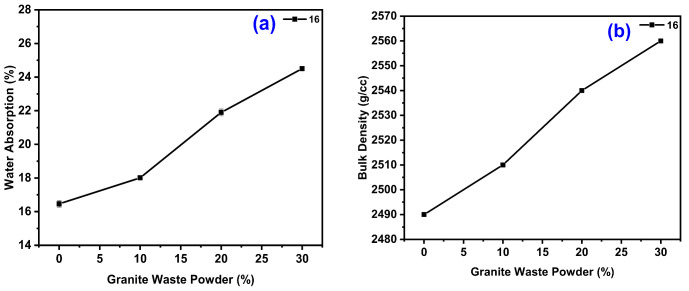
Variation of GWP on the different molarity for the WB (**a**) and BD (**b**) properties, respectively.

The WB and BD results from the changes in GWP with different molar concentrations are shown in Figure 5. For 16 M, with an A/B ratio of 0.30, the WA and BD were at a maximum for GWP with 30% content with 24% and 2560 g/cc, respectively, with minimum WA and BD for GPC with GWP of 0% (GGBS as 100%) with 16.5% and 2490 g/cc, respectively. As the molarity of the solution increases in GWP content, the WA and BD also increases.

Increases in the liquid/solid ratio result in a decrease in the activator molar and alkalinity and a decrease in its impact on the FA-GGBS matrix. However, raising the liquid/solid ratio lowers the pores in the macrostructure, resulting in denser GPC with abundant silicon compared to GGBS and GWP with a larger amount of CaO than GGBS. Geopolymer gel is created when reactive alumina reacts with an alkaline substance [18,62,71]. When the geopolymer paste is exposed to vibration during the GPC manufacturing process, the geopolymer paste bleeds, and the gel increases. The aggregate effect prevents the gel from evaporation, which results in high gel concentration in the aggregate bottom. Gel below aggregate results in reduced activator molarity and alkalinity, as well as a slower rate of polymerization, resulting in a porous binder [63,67,72]. The absorption property of GPC is reduced when the A/B ratio rises. Because the activator contains Na_2_SiO_3_, increasing the A/B ratio increases Si, resulting in a rise in SiO_2_/Al_2_O_3_ in the matrix and stronger Si-O-Si interactions [51,64,68,72].

## 5. Commercial Value and Cost Estimation

A comparative analysis was carried out to study the commercial importance of GPC in comparison to conventional concrete (Table 3). The estimation of cost analysis, based on the inclusion only of material and quantity per cubic meter, was calculated and multiplied with current market rates to estimate an approximate cost. For GWP, only charges for transportation of material were included; no charges for materials were involved. Distilled water was used to prepare GPC, while tap water was used to prepare conventional concrete. For GPC, the cost was $111.65 per m^−3^. However, for conventional concrete, it was $97.95 per m^−3^. GPC cost of materials was lower than conventional concrete. Cost estimates may still vary or become less, depending on the local availability of materials.

## 6. Conclusions

In current work, the influence of GWP on the mechanical characteristics of GGBS-based GPC was studied for different molar concentrations ranging from 12 to 18 M with a two M interval and an A/B ratio of 0.3. It was found that the slump value increases as the molar concentration increases. For 16 M of GPC, maximum slump was observed for GWP with 60 mm compared with another molar. For 16 M of GPC, maximum CS was observed for GWP with 20% with 33.95 MPa. For 16 M of GPC, maximum STS observed for GWP with 20% with 3.15 MPa. For 16 M of GPC, maximum FS observed for GWP with 20% with 4.79 MPa. The CS, STS, FS value increases with GWP content up to 20%; however, above this percentage, the performances decline. The WA and BD increase as the molar concentration of the solution increases with increased GWP concentration. Primary contents in GGBS and GWP are Si and Al, which affect the acceleration rate of geopolymerization. The rise in reactive particles implies that the alkali fuses, resulting in physicochemical changes such as crystalline phase disruption and the release of silica and alumina, which increases polymerization reactivity. A high-molecular-weight activator enhances polycondensation, resulting in a faster rate of chemical reaction in the polymer matrix. In additional to these chemical modification observations in the geopolymer, efforts have also been made to understand the economic importance and cost estimation. It was found that the geopolymer is ecofriendly, with a slightly higher cost. However, its cost mainly depends on raw material availability, which can be negotiated and, in which case, the geopolymer cost becomes closer to conventional concrete, i.e., per cubic meter, GPC has a $111.65 cost of production, while conventional concrete has an $97.95 cost. However, GPC constituent materials considered in bulk amount may be relatively closer to conventional concrete.

## Figures and Tables

**Table 1:** Chemical composition and physical properties of GGBS and GWS.

Characteristics	GGBS (wt. %)	GWS (wt. %)
Chemical Composition		
Silica	27–38	72.04%
Aluminum oxide	7–12	14.42%
Iron oxide	0.2–1.6	1.68%
Calcium oxide	34–43	1.82%
Magnesium oxide	0.15–0.76	0.71%
Titanium oxide	-	0.30%
Phosphorous	-	0.12%
Sulfates	1.0–1.9	-
Alkali oxide	-	-
Loss of ignition	1.9	0.29
Physical Properties		
Specific Gravity	2.91	2.59
Specific Surface Area	400 m^2^/kg	370 m^2^/kg

**Table 2:** Mix proportion considered for GPC.

Mix ID	GGBS %	GWP %	Fine Aggregate (kg/m^3^)	Coarse Aggregate (kg/m^3^)	NaOH (kg/m^3^)	Na_2_SiO_3_ (kg/m^3^)	Molarity (M)	Alkali/Binder Ratio	Admixture Dosage (kg/m^3^)
Standard	100	0	556	1296	14.9	52.4	12	0.30	0.0
12G0	100	0	556	1296	14.9	52.4	12	0.30	8.5
12G10	90	10	556	1296	14.9	52.4	12	0.30	8.5
12G20	80	20	556	1296	14.9	52.4	12	0.30	8.5
12G30	70	30	556	1296	14.9	52.4	12	0.30	8.5
14G0	100	0	556	1296	14.9	52.4	14	0.30	8.5
14G10	90	10	556	1296	14.9	52.4	14	0.30	8.5
14G20	80	20	556	1296	14.9	52.4	14	0.30	8.5
14G30	70	30	556	1296	14.9	52.4	14	0.30	8.5
16G0	100	0	556	1296	14.9	52.4	16	0.30	8.5
16G10	90	10	556	1296	14.9	52.4	16	0.30	8.5
16G20	80	20	556	1296	14.9	52.4	16	0.30	8.5
16G30	70	30	556	1296	14.9	52.4	16	0.30	8.5
18G0	100	0	556	1296	14.9	52.4	18	0.30	8.5
18G10	90	10	556	1296	14.9	52.4	18	0.30	8.5
18G20	80	20	556	1296	14.9	52.4	18	0.30	8.5
18G30	70	30	556	1296	14.9	52.4	18	0.30	8.5

**Table 3:** Cost analysis of GPC and conventional concrete.

Sl. No.	Materials	Quantity	Rates (INR)	Cost (USD)
Geopolymer Concrete				
1	GGBS	281.8 kg/m^3^	3000/t	845.4
2	Granite waste powder	122.7 kg/m^3^	100/t	12.27
3	Fine aggregate	554 kg/m^3^	3800/t	2105.2
4	Coarse aggregate	1294 kg/m^3^	1000/t	1294.0
5	NaOH	14.66 kg/m^3^	95/kg	1392.7
6	Na_2_SiO_3_	52.4 l/m^3^	25/kg	1310.0
7	Distilled water	43.09 l/m^3^	20/liter	861.8
8	Admixture–SP 430	4.9 l/m^3^	100/liter	491
		**Total**	**7804 ₹ m^−3^**	**111.65 $ m^−3^**
**Conventional Concrete**				
10	Cement-43 grade OPC	420 kg/m^3^	350/Bags	2940
11	Fine aggregate	700 kg/m^3^	3800/t	2660.0
12	Coarse aggregate	1200 kg/m^3^	1000/t	1200.0
13	Drinking water	150 l/m^3^	2/liter	300.0
14	Admixture–SP 430	1.89 l/m^3^	100/liter	189.0
		**Total**	**7289 ₹ m^−3^**	**97.95 $ m^−3^**

## Data Availability

Data available in a publicly accessible repository.

## References

[1] Pacheco-Torgal F., Chindaprasirt P., Ozbakkaloglu T. Handbook of Advances in Alkali-Activated Concrete.

[2] Palomo A., Maltseva O., Garcia-Lodeiro I., Fernández-Jiménez A. (2021). Portland Versus Alkaline Cement: Continuity or Clean Break: “A Key Decision for Global Sustainability”. Front. Chem..

[3] Hassan A., Arif M., Shariq M. (2020). Age-dependent compressive strength and elastic modulus of fly ash-based geopolymer concrete. Struct. Concr..

[4] Nagaraj V.K., Venkatesh Babu D.L. (2018). Assessing the performance of molarity and alkaline activator ratio on engineering properties of self-compacting alkaline activated concrete at ambient temperature. J. Build. Eng..

[5] Topçu İ.B., Toprak M.U., Uygunoğlu T. (2014). Durability and microstructure characteristics of alkali activated coal bottom ash geopolymer cement. J. Clean. Prod..

[6] Verma M., Dev N. (2021). Effect of ground granulated blast furnace slag and fly ash ratio and the curing conditions on the mechanical properties of geopolymer concrete. Struct. Concr..

[7] Haruna S., Mohammed B.S., Wahab M.M.A., Liew M.S. (2020). Effect of paste aggregate ratio and curing methods on the performance of one-part alkali-activated concrete. Constr. Build. Mater..

[8] Krishna Rao A., Kumar D.R. (2020). Effect of various alkaline binder ratio on geopolymer concrete under ambient curing condition. Mater. Today Proc..

[9] Aliabdo A.A., Abd Elmoaty A.E.M., Salem H.A. (2016). Effect of water addition, plasticizer and alkaline solution constitution on fly ash based geopolymer concrete performance. Constr. Build. Mater..

[10] Singhi B., Laskar A.I., Ahmed M.A. (2016). Investigation on soil–geopolymer with slag, fly ash and their blending. Arab. J. Sci. Eng..

[11] Ghafoor M.T., Khan Q.S., Qazi A.U., Sheikh M.N., Hadi M.N.S. (2021). Influence of alkaline activators on the mechanical properties of fly ash based geopolymer concrete cured at ambient temperature. Constr. Build. Mater..

[12] Koushkbaghi M., Alipour P., Tahmouresi B., Mohseni E., Saradar A., Sarker P.K. (2019). Influence of different monomer ratios and recycled concrete aggregate on mechanical properties and durability of geopolymer concretes. Constr. Build. Mater..

[13] Poloju K.K., Sinivasu K. (2021). Influence of GGBS and Alkaline Ratio on Compression Strength of Geopolymer Concrete: Influence of GGBS and Alkaline Ratio on Compression Strength of Geopolymer Concrete. SPAST Abstr..

[14] Kumar V.V.P., Prasad N., Dey S. (2020). Influence of metakaolin on strength and durability characteristics of ground granulated blast furnace slag based geopolymer concrete. Struct. Concr..

[15] Cao Y.-F., Tao Z., Pan Z., Wuhrer R. (2018). Effect of calcium aluminate cement on geopolymer concrete cured at ambient temperature. Constr. Build. Mater..

[16] EL Alouani S.M., Alehyen M., EL Achouri A., Hajjaji C., Taibi M. (2020). Influence of the Nature and Rate of Alkaline Activator on the Physicochemical Properties of Fly Ash-Based Geopolymers. Adv. Civ. Eng..

[17] Kantarci F., Türkmen İ., Ekinci E. (2021). Influence of various factors on properties of geopolymer paste: A comparative study. Struct. Concr..

[18] Ghannam S., Najm H., Vasconez R. (2016). Experimental study of concrete made with granite and iron powders as partial replacement of sand. Sustain. Mater. Technol..

[19] Ganesan N., Abraham R., Deepa Raj S. (2015). Durability characteristics of steel fibre reinforced geopolymer concrete. Constr. Build. Mater..

[20] Muraleedharan M., Nadir Y. (2021). Factors affecting the mechanical properties and microstructure of geopolymers from red mud and granite waste powder: A review. Ceram. Int..

[21] Menezes R.R., Ferreira H.S., Neves G.D.A., Ferreira H.C. (2002). The use of granite wastes as ceramic raw materials. Cerâmica.

[22] Gao X., Yuan B., Yu Q.L., Brouwers H.J.H. (2017). Characterization and application of municipal solid waste incineration (MSWI) bottom ash and waste granite powder in alkali activated slag. J. Clean. Prod..

[23] Al Bakri Abdullah M.M., Kamarudin H., Abdulkareem O.A.K.A., Ghazali C.M.R., Rafiza A.R., Norazian M.N. (2012). Optimization of Alkaline Activator/Fly ASH Ratio on the Compressive Strength of Manufacturing Fly ASH-BASED Geopolymer. Appl. Mech. Mater..

[24] Xie T., Visintin P., Zhao X., Gravina R. (2020). Mix Design and Mechanical Properties of Geopolymer and Alkali Activated Concrete: Review of the state-of-the-art and the Development of a New Unified Approach. Constr. Build. Mater..

[25] Thormark C. (2002). A low energy building in a life cycle—Its embodied energy, energy need for operation and recycling potential. Build. Environ..

[26] Verma M., Dev N. (2021). Sodium hydroxide effect on the mechanical properties of flyash-slag based geopolymer concrete. Struct. Concr..

[27] Ikeda K. (2007). Recent Development of Geopolymer Technique in Relevance to Carbon Dioxide and Waste Management Issues. Dev. Porous Biol. Geopolym. Ceram. Eng. Sci. Proc..

[28] Durak U., Karahan O., Uzal B., İlkentapar S., Atiş C.D. (2021). Influence of nano SiO_2_ and nano CaCO_3_ particles on strength, workability, and microstructural properties of fly ash-based geopolymer. Struct. Concr..

[29] Mehta A., Siddique R. (2017). Sulfuric acid resistance of fly ash based geopolymer concrete. Constr. Build. Mater..

[30] Deb P.S., Sarker P.K., Barbhuiya S. (2016). Sorptivity and acid resistance of ambient-cured geopolymer mortars containing nano-silica. Cem. Concr. Compos..

[31] Nuaklong P., Sata V., Chindaprasirt P. (2016). Influence of recycled aggregate on fly ash geopolymer concrete properties. J. Clean. Prod..

[32] Jayarajan G., Arivalagan S. (2021). An experimental studies of geopolymer concrete incorporated with fly-ash & GGBS. Mater. Today Proc..

[33] Mallikarjuna Rao G., Gunneswara Rao T.D. (2015). Final Setting Time and Compressive Strength of Fly Ash and GGBS-Based Geopolymer Paste and Mortar. Arab. J. Sci. Eng..

[34] (1959). IS 1199.

[35] (1959). IS 516.

[36] (1999). IS 5816.

[37] ASTM C1585-20 Standard Test Method for Measurement of Rate of Absorption of Water by Hydraulic-Cement Concretes. https://www.techstreet.com/standards/astm-c1585-20?product_id=2189851.

[38] Provis J.L., Palomo A., Shi C. (2015). Advances in understanding alkali-activated materials. Cem. Concr. Res..

[39] Bakthavatchalam K., Rajendran M. (2021). An experimental investigation on potassium activator based geopolymer concrete incorporated with hybrid fibers. Mater. Today Proc..

[40] Liew Y.-M., Heah C.-Y., Li L., Jaya N.A., Abdullah M.M.A.B., Tan S.J., Hussin K. (2017). Formation of one-part-mixing geopolymers and geopolymer ceramics from geopolymer powder. Constr. Build. Mater..

[41] Padmakar M., Barhmaiah B., Leela Priyanka M. (2021). Characteristic compressive strength of a geo polymer concrete. Mater. Today Proc..

[42] Kumar R., Das P., Beulah M., Arjun H.R., Ignatius G. (2017). Utilization of Iron Ore Tailings for the Production of Fly Ash—GGBS-Based Geopolymer Bricks. J. Adv. Manuf. Syst..

[43] Lee W.K.W., van Deventer J.S.J. (2007). Chemical interactions between siliceous aggregates and low-Ca alkali-activated cements. Cem. Concr. Res..

[44] Amran Y.H.M., Alyousef R., Alabduljabbar H., El-Zeadani M. (2020). Clean production and properties of geopolymer concrete—A review. J. Clean. Prod..

[45] Chindaprasirt P., Jaturapitakkul C., Chalee W., Rattanasak U. (2009). Comparative study on the characteristics of fly ash and bottom ash geopolymers. Waste Manag..

[46] Garcia-Lodeiro I., Palomo A., Fernández-Jiménez A., Pacheco-Torgal F., Labrincha J.A., Leonelli C., Palomo A., Chindaprasirt P. (2015). 2—An overview of the chemistry of alkali-activated cement-based binders. Handbook of Alkali-Activated Cements, Mortars and Concretes.

[47] Huseien G.F., Ismail M., Khalid N.H.A., Hussin M.W., Mirza J. (2018). Compressive strength and microstructure of assorted wastes incorporated geopolymer mortars: Effect of solution molarity. Alex. Eng. J..

[48] Samadi M., Huseien G.F., Lim N.H.A.S., Mohammadhosseini H., Alyousef R., Mirza J., Rahman A.B.A. (2020). Enhanced performance of nano-palm oil ash-based green mortar against sulphate environment. J. Build. Eng..

[49] Heah C.Y., Kamarudin H., Mohd Mustafa Al-Bakri A., Mohamed B., Luqman M., Khairul Nizar I., Liew Y.M. (2012). Effect of alkali concentration on mechanical properties of kaolin geopolymers. Rom. J. Mater..

[50] AlKhatib A., Maslehuddin M., Al-Dulaijan S.U. (2020). Development of high performance concrete using industrial waste materials and nano-silica. J. Mater. Res. Technol..

[51] Xie J., Chen W., Wang J., Fang C., Zhang B., Liu F. (2019). Coupling effects of recycled aggregate and GGBS/metakaolin on physicochemical properties of geopolymer concrete. Constr. Build. Mater..

[52] Yu L., Li Y., Liu T., Qin Z., Tan H., Zhang H., Chen Z., Ni H. (2021). Mechanical and microstructural characterization of geopolymers synthesized from FCC waste catalyst and silica fume. Ceram. Int..

[53] Their J.M., Özakça M. (2018). Developing geopolymer concrete by using cold-bonded fly ash aggregate, nano-silica, and steel fiber. Constr. Build. Mater..

[54] Ganesh A.C., Muthukannan M. (2021). Development of high performance sustainable optimized fiber reinforced geopolymer concrete and prediction of compressive strength. J. Clean. Prod..

[55] Luhar S., Chaudhary S., Luhar I. (2019). Development of rubberized geopolymer concrete: Strength and durability studies. Constr. Build. Mater..

[56] Okoye F.N., Prakash S., Singh N.B. (2017). Durability of fly ash based geopolymer concrete in the presence of silica fume. J. Clean. Prod..

[57] Pasupathy K., Berndt M., Sanjayan J., Rajeev P., Cheema D.S. (2017). Durability of low-calcium fly ash based geopolymer concrete culvert in a saline environment. Cem. Concr. Res..

[58] Sethi H., Bansal P.P., Sharma R. (2019). Effect of Addition of GGBS and Glass Powder on the Properties of Geopolymer Concrete. Iran. J. Sci. Technol. Trans. Civ. Eng..

[59] Bernal S.A., Mejía de Gutiérrez R., Pedraza A.L., Provis J.L., Rodriguez E.D., Delvasto S. (2011). Effect of binder content on the performance of alkali-activated slag concretes. Cem. Concr. Res..

[60] Yip C.K., Lukey G.C., Provis J.L., van Deventer J.S.J. (2008). Effect of calcium silicate sources on geopolymerisation. Cem. Concr. Res..

[61] Chithambaram S.J., Kumar S., Prasad M.M., Adak D. (2018). Effect of parameters on the compressive strength of fly ash based geopolymer concrete. Struct. Concr..

[62] Singh B., Ishwarya G., Gupta M., Bhattacharyya S.K. (2015). Geopolymer concrete: A review of some recent developments. Constr. Build. Mater..

[63] Chindaprasirt P., Chalee W. (2014). Effect of sodium hydroxide concentration on chloride penetration and steel corrosion of fly ash-based geopolymer concrete under marine site. Constr. Build. Mater..

[64] Hanjitsuwan S., Hunpratub S., Thongbai P., Maensiri S., Sata V., Chindaprasirt P. (2014). Effects of NaOH concentrations on physical and electrical properties of high calcium fly ash geopolymer paste. Cem. Concr. Compos..

[65] Malkawi A.B., Nuruddin M.F., Fauzi A., Almattarneh H., Mohammed B.S. (2016). Effects of Alkaline Solution on Properties of the HCFA Geopolymer Mortars. Procedia Eng..

[66] Mousavinejad S.H.G., Gashti M.F. (2021). Effects of alkaline solution/binder and Na_2_SiO_3_/NaOH ratios on fracture properties and ductility of ambient-cured GGBFS based heavyweight geopolymer concrete. Structures.

[67] Nuaklong P., Jongvivatsakul P., Pothisiri T., Sata V., Chindaprasirt P. (2020). Influence of rice husk ash on mechanical properties and fire resistance of recycled aggregate high-calcium fly ash geopolymer concrete. J. Clean. Prod..

[68] Nuaklong P., Wongsa A., Boonserm K., Ngohpok C., Jongvivatsakul P., Sata V., Sukontasukkul P., Chindaprasirt P. (2021). Enhancement of mechanical properties of fly ash geopolymer containing fine recycled concrete aggregate with micro carbon fiber. J. Build. Eng..

[69] Duxson P., Provis J.L., Lukey G.C., van Deventer J.S.J. (2007). The role of inorganic polymer technology in the development of ‘green concrete’. Cem. Concr. Res..

[70] Görhan G., Kürklü G. (2014). The influence of the NaOH solution on the properties of the fly ash-based geopolymer mortar cured at different temperatures. Compos. Part B Eng..

[71] Prinsse S., Hordijk D.A., Ye G., Lagendijk P., Luković M. (2020). Time-dependent material properties and reinforced beams behavior of two alkali-activated types of concrete. Struct. Concr..

[72] Bouaissi A., Li L., Al Bakri Abdullah M.M., Bui Q.-B. (2019). Mechanical properties and microstructure analysis of FA-GGBS-HMNS based geopolymer concrete. Constr. Build. Mater..

